# Medical Department of Cincinnati College

**Published:** 1835

**Authors:** 


### Art. II. — The Medical Department of the Cincinnati College.

The July number of our Journal, set forth, in its appendix, an account of the organization, in this city, of a new Medical School, under the auspices of the Cincinnati College. It went into operation at the time proposed, and the delay in the publication of the present number, enables us to introduce, as a supplement, the first catalogue of students, and, also, a list of the graduates at the first Commencement, which has just taken place. The former will be found to be sixty-six, and the latter eighteen.* The same supplement contains a notice of the future sessions of the school; and of a summer Institute, to which most of the professors belong.

Connected as we are with these projects, it does not become us to pronounce decisively on the omen which the success already attained affords, but we cannot resist the opinion, that the prospects of the new school are highly auspicious. If we mistake not, there has not, before, been so numerous a class, in the opening session of any other medical institution in the Union, nor one made up from so many different quarters. The first class of Transylvania University was twenty; the second thirty-eight; the third but ninety-three: —The first class of the Medical College of Ohio was twenty-five, the second thirty, and it required five years more to enable it to reach the number with which the Cincinnati College has begun.

* Of these eighteen, three had been practitioners for four years, two had attended a course of lectures in the Philadelphia schools, and thirteen had been pupils in the Medical College of Ohio.

It was not till the latter part of the month of May of the last year, that the Medical Department of this Institution was projected. Professors were then to be obtained, a decaying edifice repaired, apparatus provided, and the medical public apprised of the undertaking. Each of these objects was beset with difficulties, but all except the last were, successfully, executed. To give adequate publicity to the enterprise, over a region of such vast extent as that from which pupils for our western schools must be derived, was found to be quite impracticable; and, it is well known, that many students arrived in Cincinnati, on their way to other schools, before they ever heard of the Cincinnati College. When they came to this knowledge, they discovered, moreover, that a majority of the professors had not belonged to any medical school, and it was of course impossible for them to know, in what manner these new professors would acquit themselves. We are happy to be able to say, that all who have thus appeared before the public for the first time, have, as far as we can learn, discharged their respective duties, to the admiration of their pupils, and that they fairly promise to become distinguished teachers.

The great departments of Anatomy—special, surgical, general, and pathological,—and of Physiology, confided to two professors, are already well supplied with preparations both healthy and morbid; the number of subjects for dissection has amounted to twenty-one, and, if more had been required, there would have been no difficulty in obtaining them. The cost to the students was eight dollars each. The chemical laboratory is, also, furnished with all the variety of apparatus required for a complete course of general and pharmaceutic chemistry. These different departments are supplied with amphitheatres of the best construction, and capacious enough to receive, each, 300 students. We have spoken especially of the arrangements made for the benefit of these professorships, because, of the whole they require the greatest amount of preparation, and are most inquired after by students of medicine; but we may say of the others, that they are provided with the requisite means of illustration; and that, in the whole Faculty, there is a spirit of activity and perseverance which cannot be quenched by ordinary causes, and will stimulate them to unwearied exertion.

